# Why We Are Losing the War Against COVID-19 on the Data Front and How to Reverse the Situation

**DOI:** 10.2196/20617

**Published:** 2021-05-05

**Authors:** David Prieto-Merino, Rui Bebiano Da Providencia E Costa, Jorge Bacallao Gallestey, Reecha Sofat, Sheng-Chia Chung, Henry Potts

**Affiliations:** 1 Faculty of Epidemiology & Population Health London School of Hygiene & Tropical Medicine London United Kingdom; 2 Applied Statistical Methods in Medical Research Group Catholic University of San Antonio in Murcia Murcia Spain; 3 Institute of Health Informatics University College London London United Kingdom; 4 University of Medical Sciences Havana Cuba

**Keywords:** COVID-19, learning health systems

## Abstract

With over 117 million COVID-19–positive cases declared and the death count approaching 3 million, we would expect that the highly digitalized health systems of high-income countries would have collected, processed, and analyzed large quantities of clinical data from patients with COVID-19. Those data should have served to answer important clinical questions such as: what are the risk factors for becoming infected? What are good clinical variables to predict prognosis? What kinds of patients are more likely to survive mechanical ventilation? Are there clinical subphenotypes of the disease? All these, and many more, are crucial questions to improve our clinical strategies against the epidemic and save as many lives as possible. One might assume that in the era of big data and machine learning, there would be an army of scientists crunching petabytes of clinical data to answer these questions. However, nothing could be further from the truth. Our health systems have proven to be completely unprepared to generate, in a timely manner, a flow of clinical data that could feed these analyses. Despite gigabytes of data being generated every day, the vast quantity is locked in secure hospital data servers and is not being made available for analysis. Routinely collected clinical data are, by and large, regarded as a tool to inform decisions about individual patients, and not as a key resource to answer clinical questions through statistical analysis. The initiatives to extract COVID-19 clinical data are often promoted by private groups of individuals and not by health systems, and are uncoordinated and inefficient. The consequence is that we have more clinical data on COVID-19 than on any other epidemic in history, but we have failed to analyze this information quickly enough to make a difference. In this viewpoint, we expose this situation and suggest concrete ideas that health systems could implement to dynamically analyze their routine clinical data, becoming learning health systems and reversing the current situation.

## The Problem

Many countries reacted late to the spread of the COVID-19 pandemic, although once they realized the seriousness of the situation, they took strong measures. The best-known measures relate to restrictions on population movement; other important implementations include increasing the capacity of health systems and the mobilization of the military to aid in this health emergency. Using a martial simile, it appears that governments have prepared their “health” armies and their populations for the war against the virus.

An additional necessity is a good intelligence service to fight the war. This requires a system to collect data on the enemy and a group of analysts who can extract relevant information. Most current information systems pertaining to the pandemic focus on counting numbers of individuals tested, infected, hospitalized with serious conditions, recovered, and deceased. Data have also been collected to understand public behaviors [1], some of which was planned for [2]. These kinds of data can serve to estimate epidemiological curves and predict how the pandemic might evolve (if everything continues as it has been so far), but they provide very limited insight into how frontline doctors can fight the virus.

Epidemiological data often combine a limited number of variables for substratification of cases; these data categorize continuous variables for reporting purposes and often lack detailed information on variables collected during hospital care. Epidemiological curves do not allow us to answer clinical questions such as what the most relevant risk factors are for becoming infected, having symptoms, becoming seriously ill, or dying. They also do not allow us to study which treatments work better and what patient characteristics can influence the success or failure of the treatments. These are the questions that we need to answer to improve patient care and to rationalize the use of resources when health systems are at the limit of their capacities. These questions have not been answered satisfactorily. On March 24, 2020, a senior intensive care unit physician working in a big hospital in Madrid told the lead author that, “We learn things about the disease as we go along every day.” Two weeks later, on April 9, 2020, a colleague working at a hospital affiliated with University College London Hospitals said, “…is a new disease with a pathology and clinical course that none of us know about.” In between these two statements, thousands of patients have died or recovered from SARS-CoV-2 infection in Spain, the United Kingdom, and many other countries. It seems we hardly learned anything from those patients since months later we continue to ask the same clinical questions: what are the determinants for bad prognosis? How do we best treat patients?

## What Do We Need to Solve the Problem?

To answer clinical questions, we need clinical data from individual patients. We need a database where the anonymous clinical information of hospitalized patients with COVID-19 can be stored, curated, and made accessible to researchers. The structure of the data set does not have to be very complex since, for each patient, the disease involves basically a single hospital episode that does not usually last more than 4-5 weeks. The database would be continually fed with each hospital discharge. Statistical models to answer each clinical question can be programmed and automatically updated as more data come in. In this way, we would have a continuous information system growing with the epidemic and generating knowledge in real time that could be fed back to the frontline doctors treating patients. Therefore, the health system to fight the epidemic would have two subsystems working together: a care subsystem to treat patients and a knowledge subsystem to learn about the disease. This is what the literature has described as a learning health system [3,4].

Although it would be ideal to link up with the patient’s medical history in specialized and primary care, this may not be necessary to answer the most pressing clinical questions about prognosis and the best therapeutic strategies. Initially, each health system (country/nation/region) would implement its own database including as many hospitals as possible although sharing information between countries would be advantageous. In addition, because the epidemic develops asynchronously in different countries, what we can learn from the data in one country can help to improve patient treatment in other countries.

## What Is Being Done?

There are many private initiatives to create registries of patients with COVID-19 that include clinical data, some supported by professional societies [5-14]. Although understandable and praiseworthy, these initiatives are generally burdened with problems:

1. Often these registries are disconnected from the electronic health records of hospitals and require extra effort from health care professionals to input the data. This has long been known to be a major barrier to usage [15]. These professionals are likely to be heavily overloaded with work, and many of them simply refuse to fill out another form.
2. Since some of these registries are for patients with a specific disease (eg, atopic dermatitis) and they try to collect very detailed patient data, entry forms can be lengthy and detailed.
3. Since some of these data sets recruit only specific kinds of patients, their analysis will only apply to certain subpopulations.
4. Contribution to these data sets is voluntary, and the speed at which they grow depends on many factors, such as how many professionals they have managed to reach and persuade to collaborate, how many patients with the specific selection criteria are available, and how easy they are to feed. For example, as of April 11, 2020, the LEOSS (Lean European Open Survey on SARS-CoV‑2 infected patients) [8] reported having 642 collaborators involved from 165 centers, but only 770 patients enrolled. However, the Intensive Care National Audit & Research Centre [6], a long-standing registry, reported having captured 2621 COVID-19 admissions to the intensive care unit by April 4, 2020.
5. Many of these registries have been designed to collect data that will be analyzed when the epidemic is over, and the research questions behind them are not always connected to the needs of health providers at the front line. Many of these analyses will not be timely enough to confront the current epidemic, and it is unclear whether they will be useful to deal with the next one. One cannot blame clinicians treating patients if they do not put much interest in filling these forms that will not result in immediate tangible benefits to their patients. Those who do the work need to see the benefits to be motivated to participate [16-18].

Some governments and health institutions have implemented important initiatives to share individual patient clinical data on COVID-19. For example, the Mexican government, following a policy of open data, has been sharing clinical data on all COVID-19 cases since April 13, 2020, with a daily update of the entire data set [19]. The Health Insurance Review & Assessment Service of South Korea also intended to make anonymized clinical data pertaining to patients with COVID-19 available [20]. One of the most comprehensive initiatives is the OpenSAFELY project [21] in the United Kingdom, which created a new secure analytics platform for electronic health records in the National Health Service (NHS) to deliver urgent results during the global COVID-19 emergency. This platform includes not only patients with COVID-19 but almost any patient registered in participating primary care practices (more than 24 million patients’ full pseudonymized primary care NHS records, with more to follow). Their complete analytic software is open for security review, scientific review, and reuse.

Although all these initiatives are laudable and valuable for facilitating important research to be undertaken and even breakthroughs to be made in the fight against the pandemic, none of them are learning systems that are integrated within health care systems. They have not been designed within the health care system to answer specific health care questions. In our view, a health learning system would work best when integrated with the care services through a constant feedback loop to respond as quickly as possible to the most pressing clinical questions as mentioned above [22]. The OpenSAFELY platform is aiming to comply with this model although it was not actually created within the NHS as an integral part of it but by academics in universities who saw the potential of making these data available to researchers outside the NHS who were eager to help in the fight against the pandemic.

## What Can Be Done?

The health systems should plan the collection, availability, analysis, and reporting of clinical data in a timely manner that can effectively influence the response to the epidemic. That is, they should strive to become learning health systems during the epidemic. We offer some suggestions to achieve this.

### 1. Design

There should be protocols to ensure that at least a minimum set of relevant clinical variables are collected using the same criteria and procedures across all units of a health system. Failure to regulate and standardize clinical data collection might lead to the development of several independent data sets that are not easy to link to one another. Interoperability solutions are vital [23]. It is not our purpose here to propose a specific set of variables for the COVID-19 pandemic; we believe this should be agreed upon by appointed expert panels, but below we point out some important considerations that should be addressed when designing the database.

#### Scope

The database should be designed in such a way that it can be implemented in most health units expected to collect the data. In general, the more heterogeneous the data collectors, the simpler the database will need to be to maximize the possibility of collecting comparable data across units. For example, if a supranational organization such as the World Health Organization (WHO) were to suggest a data structure, it is advisable to keep the specifications as simple as possible to be applicable to most countries, bearing in mind the diversity of health systems and resources available. In contrast, a country with a highly digitalized and homogeneous health system can specify a more complex data structure. The advantages of the WHO approach would be to potentially collect a much larger data set covering diverse populations in different countries, while a country-specific model could collect less but more detailed data. A combination of the two approaches might be possible with the WHO producing a data structure with different layers of complexity starting from a top, simpler level collecting the most basic data to answer the most pressing questions down to levels of highly complex data. Each health system could then try to achieve as many levels as possible depending on their capabilities.

#### Objectives

It is important to design the data collection process with a specific research question(s) in mind to determine how and what data should be collected. For example, to answer the question “what risk factors affect the probability of infection?” we might want to collect information on the patient’s circumstances (social, family, and work) before and around the infection time, but to answer the question “what conditions determine poor progression after hospital admission?” it might be enough to collect clinical information at hospital admission. A question about treatment effectiveness will require health services to collect detailed information on treatments and all potential confounding factors. In general, the more questions we want to answer, the more data we need to capture and the more complex the system will be.

#### Costs

Obtaining each piece of information bears a cost. Sometimes this is minimal (eg, when information is collected routinely in the health system and easily retrievable) or it can require specialized resources and personnel who might not be available. Costly and complex information is more likely to not be properly collected in a system already under stress. Information partially and poorly collected is a potential source of bias if included in the analysis, and it might be therefore better to ignore it altogether. Hence, the costs and benefits of the information must be carefully balanced to reduce waste of resources, time, and the potential for bias.

#### Benefits

The benefit of variables is their potential contribution to answer one or more of the research questions. This does not mean that we can only include in the database variables that we know in advance to have some explanatory power for the outcomes that we want to study; in fact, we will not know this until we analyze the data after collection. However, there must be some reasonable expectations that the variable has an explanatory role or is a potential confounder that we need to adjust for in the analysis. The potential benefits must outweigh the costs of collecting and processing the information. Collecting variables with a priori limited expected explanatory power just in case someone finds a good use for them in the future is not a wise strategy when answers need to be found quickly. Apart from the extra cost of collection and processing, more statistical models might need to be run to confirm whether those variables are indeed irrelevant. This can unnecessarily increase the chances of finding false-positive associations that might divert efforts and attention from exploring the important causal associations [24].

In summary, a combined panel of experts with knowledge of the specific disease, epidemiology, health information systems, and statistics should design a database structure to answer a specific set of questions, considering the cost/benefit balance of the information to be collected, bearing in mind who is going to collect the data, and possibly proposing several layers of data collection from a minimum, simpler-to-collect set of variables to more complex data structures for highly digitalized systems.

### 2. Plan

A good plan will fail if the means to execute it are not made available. Technology can facilitate the collection of data. Where good electronic health records exist, relevant clinical data can be extracted from them. The particular implementation will depend on the characteristics of each health system and the data to be collected as defined in the *Design* section. For example, if a health system is highly informatized and most of the required data are already collected in electronic health records routinely, then perhaps only a database operator is needed to extract the relevant data. If the health system’s information is mainly on paper, a team of dedicated researchers with a sufficient medical background will need to be recruited to extract the data and input the information into an electronic format. We cannot specify here what kind of professionals need to be hired or reinforced in each health system, but we can provide some points that should be considered to define needs:

- Consider the level of medical/epidemiological knowledge needed for the personnel involved in data extraction;
- Consider the technical difficulties of data extraction, storage, and curation, as well as knowledge of information technology and informatics needed to do this;
- Consider the complexity of the statistical analyses to be done with the data.

The golden rule is that these processes should not burden and distract care providers from their main job—treating the patients. Similar to how extra teams of doctors and nurses were brought in to care for patients on the frontline, and mathematical modelers were recruited to predict the progression of the epidemic, experts in medical informatics and data analysis should also be recruited to help analyze the clinical data on a nearly daily basis.

### 3. Integrate

Protocols permitting the two subsystems, care provision and data analysis, to work in synchrony need to be established [22]. The care system will provide data and the relevant questions, and the analysis system will run statistical and computer models to try to answer those questions. The findings will then be fed back to the care system, which can revise its strategy in light of new evidence. In the initial stages of the epidemic, clinical questions can be formulated, and the analysis team can design Bayesian models using existing prior clinical and biological knowledge. Once data start accumulating, the Bayesian models will be updated, and they can be used for decision making.

The kind of study designs that can be implemented in a learning health system can vary from simple case reports to randomized clinical trials, and one can potentially do several designs in parallel [25]. For example, a cohort analysis can be set up to look at risk factors at the diagnosis point for disease progression, so that patients can be triaged more efficiently at overloaded hospital admission departments. At the same time, if clinicians had clinical equipoise of treatment options for a subgroup of patients, a registry-based randomized clinical trial could be set up to decide the best treatment options in a timely manner [26-28]. In large health systems that cover most of the population, cases can be captured rather quickly if the learning system is implemented and coordinated across sites (hence the need for regulating and standardizing the methods).

### 4. Enforce

Data collection should not be regarded as an optional task in an epidemic as it is a necessary resource to learn how to fight the epidemic more effectively, to guarantee the right of the patient to the best possible care, and to reduce the number of deaths. The term “enforcement” might have negative connotations when related to health care research where we expect that participation of both researchers and patients should always be voluntary. However, a pandemic is an exceptional situation where the health (and possibly survival) of the population is at serious risk, and the public good must be balanced against individual rights. This is indeed the case when restrictions such as quarantines or curfews are imposed during pandemics. Enforcement is much more likely to work if care professionals and patients are willing to collaborate. Early engagement through appropriate communication with health professionals and the public is explained below.

### 5. Engage Health Professionals

Collaboration as early as possible with clinical users is key to ensure these systems are usable and useful and to encourage adoption [3,18,23]. Cooperation of clinical users is not only key to ensuring good data quality; they also have to produce relevant clinical questions and incorporate the new knowledge generated into their practice. For care providers to spend time and energy engaging with the system, they must see it as an investment that will benefit their work and their patients rather than another administrative burden. It is crucial that they are involved in the design of the system to ensure that the system meets their needs and to facilitate trust in the system.

### 6. Inform the Population

Plans need to be made to address concerns around patient privacy and confidentiality [23], including appropriate legislative frameworks for emergency situations. In particular, ethical issues around data usage for research, by people inside and outside the system, need to be tackled from the beginning. In a setting of an infectious epidemic, it might be impossible to obtain informed consent from every single patient for their data to be used for research purposes. There are also procedures by which a priori one presumes consent of a patient who is unable to provide it in a critical situation, and later this consent is confirmed when the patient recovers or through relatives, and the data can be pulled out of the study if consent is revoked. These procedures have been implemented successfully in randomized clinical trials of emergency treatments [29].

On the other hand, these data are urgently needed to learn as quickly as possible about the disease, to stop the epidemic, and to save lives. The right to confidentiality should be balanced against urgency to control the epidemic. Data for research should be anonymized as much as possible. Ideally, an anonymized data set would have the ethical approval to be freely used for research in such a way that individual researchers would not have to seek approval separately for each project that uses the same data.

### 7. Share

Data should be made available to external researchers, and administrative burden should be reduced. Often, research projects in traditional settings have to go through tedious administrative procedures, seeking approval from different committees on different aspects such as ethics, technical quality, economic viability, chances of success, etc. This can delay data acquisition, analysis, and generation of results by weeks or months that might cost thousands of lives during an epidemic. Within a learning health system that sets up its own analysis teams, which are well coordinated with clinical teams, as specified in the *Plan* section, most of these requirements should be removed, so that the analysis can be done efficiently in real time in house. However, the system should also consider opening its data (anonymized as required) to external researchers. The health system could then benefit from the brain power of thousands of teams that might be able to tackle many problems simultaneously from different angles. For example, in the current COVID-19 epidemic, there have been thousands of highly qualified researchers literally locked at home, willing to help, and eager to analyze clinical data. Health systems might not have the capacity and resources to recruit, incorporate, and coordinate all these groups into their internal structure, but they might benefit from their ideas and skills if they provided them with data and research questions. This could render benefits to the health system, the scientific community, and society as a whole. However, to allow these groups to provide answers quickly, administrative procedures should be simplified as much as possible. For example, if adequately anonymized data sets are created and approved for external research by an ethics committee of the health system, they can be made freely accessible and readily usable to external researchers without having each of them apply and wait for ethics approval.

In the current COVID-19 epidemic, most of the registries mentioned above have tried to implement this model. However, to make the data accessible, they still require the potential researcher to present a project that has to be evaluated by an expert committee before the data can be released. The consequence of this policy is to produce a bottleneck of project approvals that delays necessary research. Paradoxically, the more relevant the data of the registry and the larger the potential research community, the bigger the bottleneck is likely to be (unless more resources are put into place to manage requests). Is not always clear what the purpose of this step is. New ethical approval should not be necessary if the data are correctly anonymized and ethically approved already. Often the argument is to act as a gatekeeper against potential “bad science” practices. However, the gate keeping can be done a posteriori by the scientific community by looking at the outputs of the research, as it is normally done for peer-reviewed publication or preprints. This will not delay the finding of potentially important results. It would certainly be encouraged to avoid publication bias by making all research available even if results are not positive or conclusive, but this can be solved through setting up a registry of protocols (similar to, for example, ClinicalTrials.gov [30]) that does not create a bottleneck neck and delay research.

### 8. Revise and Update

A health learning system is not a one-off exercise in design. It should be a life system constantly adapting itself to a changing environment [22], especially during epidemics where the situation is expected to change rapidly. There should be a permanent committee made up of clinicians, epidemiologists, health care system experts, medical informatic experts, and statisticians, who will supervise (daily if needed) the functioning of the system and steer the necessary changes. The committee should check, on a long-term basis, if:

- The system is doing what it was designed to do, that is, collect the right data, perform the planned analysis, and answer the questions that were asked);
- The outcomes from the learning system are having an impact on health care decisions and outcomes (ie, assess whether there is a connection between the two systems);
- Any new questions need to be answered as the epidemic evolves. If yes, determine how the learning system should be adapted to address them.

## Conclusions

This paper is a reflection on the lack of a strategy involving learning health systems, which is proving to be a critical shortcoming of the current health care systems’ ability to fight the pandemic. It is also a proposal of points that need to be considered for implementation and integration, such as learning systems within the health care system. Different countries may need to apply different strategies to organize and implement a system to collect, analyze, and disseminate results and relevant information in a timely fashion. Those strategies will depend on health governance, and, in particular, on how health systems are structured around a unique legal and administrative body.

However, as a scientific community, we need to change the way we think about clinical data and clinical research in epidemics. Clinical data should be valued not only as an information source for the patient who has generated it but also as the main resource for learning about the disease and saving the next patient. Clinical research should not be considered an academic activity to be done once the epidemic is over; it should be viewed as the main way of learning from clinical data and be completed as close as possible in time during clinical practice while the epidemic is ongoing and with the fastest possible feedback between the two activities (care and research).

Perhaps the most compelling evidence that we are doing things wrong is the actual macro figures of the COVID-19 epidemic. As of today, with over 117 million confirmed cases around the world and deaths approximating the 3-million mark, we are still asking many of the clinical questions that we were asking in the beginning. We hope that this piece acts as a wake-up call on this issue.

## Abbreviations

LEOSS: Lean European Open Survey on SARS-CoV‑2 infected patients
NHS: National Health Service
WHO: World Health Organization
